# The protein kinase MBK-1 contributes to lifespan extension in *daf-2* mutant and germline-deficient *Caenorhabditis elegans*

**DOI:** 10.18632/aging.101244

**Published:** 2017-05-25

**Authors:** Hildegard I. D. Mack, Peichuan Zhang, Bryan R. Fonslow, John R. Yates

**Affiliations:** 1 Department of Biochemistry and Biophysics, University of California, San Francisco, San Francisco, CA 94158, USA; 2 Department of Chemical Physiology, The Scripps Research Institute, La Jolla, CA 92037, USA; 3 Present address: Institute for Biomedical Aging Research, Leopold-Franzens-Universität Innsbruck, Innsbruck 6020, Austria; 4 Present address: Calico Life Sciences, South San Francisco, CA 94080, USA

**Keywords:** FOXO, DYRK1, aging, phosphorylation, signaling

## Abstract

In *Caenorhabditis elegans*, reduction of insulin/IGF-1 like signaling and loss of germline stem cells both increase lifespan by activating the conserved transcription factor DAF-16 (FOXO). While the mechanisms that regulate DAF-16 nuclear localization in response to insulin/IGF-1 like signaling are well characterized, the molecular pathways that act in parallel to regulate DAF-16 transcriptional activity, and the pathways that couple DAF-16 activity to germline status, are not fully understood at present. Here, we report that inactivation of MBK-1, the *C. elegans* ortholog of the human FOXO1-kinase DYRK1A substantially shortens the prolonged lifespan of *daf-2* and *glp-1* mutant animals while decreasing wild-type lifespan to a lesser extent. On the other hand, lifespan-reduction by mutation of the MBK-1-related kinase HPK-1 was not preferential for long-lived mutants. Interestingly, *mbk-1* loss still allowed for DAF-16 nuclear accumulation but reduced expression of certain DAF-16 target genes in germline-less, but not in *daf-2* mutant animals. These findings indicate that *mbk-1* and *daf-16* functionally interact in the germline- but not in the *daf-2* pathway. Together, our data suggest *mbk-1* as a novel regulator of *C. elegans* longevity upon both, germline ablation and DAF-2 inhibition, and provide evidence for *mbk-1* regulating DAF-16 activity in germline-deficient animals.

## INTRODUCTION

FOXO transcription factors are evolutionarily conserved regulators of cell proliferation, differentiation, survival and metabolism and play a key role in maintaining cellular homeostasis, particularly under stress conditions [1]. On the organismal level, FOXO orthologs modulate lifespan in a broad variety of species, e.g. in the nematode *Caenorhabditis elegans*, the fruit fly *Drosophila melanogaster* and, possibly, in mice, where FOXO family members also have been implicated in age-related diseases such as cancer and type 2 diabetes [1–5]. Interestingly, several studies indicate that polymorphisms in the human *FOXO3A*-gene are positively associated with longevity in both genders, while one study also found a negative association of *FOXO1A*-variants with longevity in women [2, 6–8].

In *C. elegans,* the sole FOXO ortholog, DAF-16, promotes longevity in response to various inputs such as decreased activity of the insulin/IGF1-like receptor DAF-2 or increased signaling through the stress-sensing AMPK-, JNK- and SIR2-pathways [9–12]. In addition, the developmental timing micro-RNA LIN-4 and the ablation of germline stem cells can activate DAF-16 and extend lifespan [13, 14]. On the molecular level, subcellular localization, stability and transcriptional activity of FOXOs are tightly regulated by post-translational modifications (PTMs) such as phosphorylation, acetylation, ubiquitylation and methylation [15]. Most of the currently known FOXO-PTMs have been identified in one of the four mammalian FOXOs, FOXO1, -3, -4 and -6, but the affected residues and the modifying enzymes are frequently conserved across species [15]. Once activated, DAF-16 extends lifespan through inducing or suppressing the expression of many genes encoding, for example, detoxifying enzymes, antimicrobial peptides, chaperones and apolipoproteins [16]. In many contexts, other transcription factors such as HSF-1 and SKN-1/Nrf2 act in concert with DAF-16 to increase lifespan [2, 17].

Germline ablation extends lifespan not only in wild type, but also in *daf-2* mutant animals, suggesting that DAF-16 activation and/or function differs between the germline- and the *daf-2* longevity pathway [13]. Indeed, reduced activity of the DAF-2/PI3-kinase/AKT pathway promotes nuclear accumulation of DAF-16 in multiple tissues and at all developmental stages [18–20]. In contrast, when germline precursor cells are ablated from L1 larvae, nuclear accumulation of DAF-16 occurs predominantly in the intestine, starts only in early adulthood, and requires the adaptor protein KRI-1 and the nuclear hormone receptor DAF-12 [18, 21, 22]. Of note, nuclear accumulation of DAF-16 is not sufficient to increase *C. elegans* lifespan, suggesting the existence of additional pathways that directly regulate DAF-16 transcriptional activity [18, 23].

*C. elegans* MBK-1 (*Drosophila melanogaster* Minibrain-related kinase) is a member of the evolutionarily conserved DYRK-family of protein kinases and orthologous to human DYRK1A/B [24]. *DYRK1A* is located in the Down syndrome critical region on chromosome 21 and has been associated with the neurological defects seen in this disease [24, 25]. Through phosphorylation of substrates on serine and threonine residues, DYRK1A/B control various cellular processes, such as cell cycle progression, differentiation and survival [24, 25]. In *C. elegans mbk-1* overexpression results in chemotaxis defects while genetic inactivation causes no obvious abnormalities [26]. Yet, there is evidence for MBK-1 being required for resistance to certain pathogens [27]. GFP-reporter studies indicate that *mbk-1* is expressed in all somatic tissues throughout development and adulthood and localizes to the nucleus in all cells [26]. In addition to MBK-1, two other DYRK family members have been described in *C. elegans*, MBK-2 (DYRK2/3) and the more distant relative HPK-1 (HIPK2) [26]. Loss of *hpk-1* has been shown previously to shorten lifespan of wild-type and of *daf-2(-)* worms [28].

Here, we report that in *C. elegans*, loss of *mbk-1* shortens the lifespan of long-lived *daf-2* and *glp-1* (germline-deficient [29]) mutant animals, while affecting the lifespan of wild-type worms to a lesser extent. Moreover, we provide evidence for *mbk-1* contributing to upregulation of some DAF-16 target genes in the *glp-1*, but not in the *daf-2* mutant background. Thus, our findings identify MBK-1 as a novel regulator of lifespan that may function differently in the germline- and in the *daf-2* longevity pathways.

## RESULTS

### Evidence for DAF-16 Ser326 phosphorylation *in vivo*

**Figure 1:**
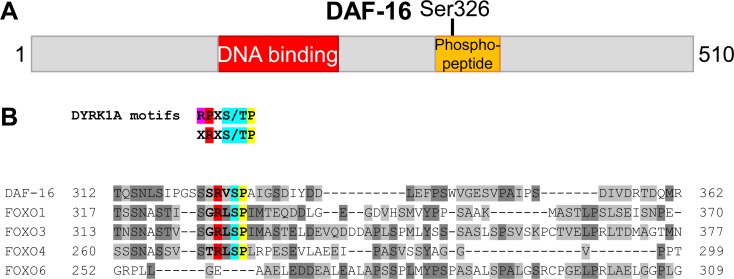
Evidence for phosphorylation of Ser326 in *C. elegans* DAF-16 (**A**) Schematic drawing (to scale) of the DAF-16 protein (isoform c/a1). The location of a phosphopeptide derived from immunoprecipitated GFP::DAF-16 by tryptic digest, is shown in orange. The phosphorylation site was mapped to Ser326. (**B**) ClustalΩ alignment of the full length sequences of human FOXO family members and *C. elegans* DAF-16. Only the part spanning the Ser326-containing phosphopeptide is shown. The phosphorylated Serine in DAF-16 (Ser326), and its corresponding sites in FOXO1 (Ser329), FOXO3 (Ser325), FOXO4 (Ser273) and FOXO6 (not present) are highlighted in blue. Additional residues specifying the DYRK1A consensus motifs [32, 33, 65] are highlighted in red and yellow.

In order to investigate how DAF-16 activity in the intestine is regulated by phosphorylation in different longevity pathways, we used mass spectrometry to analyze immunoprecipitates of intestinally expressed GFP::DAF-16 (encoded by transgene *muIs194, daf-16* isoform c, also known as isoform a1) from lysates of three different strains: (1) *daf-16(mu86), muIs194* (referred to as wild-type in the context of mass spectrometry experiments), (2) *daf-16(mu86); daf-2(e1370); muIs194* (referred to as *daf-2* mutant), and (3) *daf-16(mu86); glp-1(e2144ts); muIs199* (referred to as *glp-1* mutant). We identified a phosphopeptide spanning Ser326 in a sample from wild-type worms. ClustalΩ alignments mapped this phosphopeptide to a region downstream of the DNA-binding (forkhead) domain (Figure 1A) and revealed that Ser326 corresponds to Ser329 in human FOXO1 and to Ser326 in murine FOXO1, previously described phosphorylation sites for the mammalian kinases DYRK1A and NLK, respectively [30, 31]. Additional sequence analysis indicated that the residues surrounding Ser326/Ser329 are well conserved between DAF-16 and FOXO1/3/4 and match the DYRK target motif RX_1-2_S/TP [32, 33] (Figure 1B). On the other hand, NLK-regulation of murine FOXO1 apparently involves concurrent phosphorylation of Ser326 and up to seven additional S/TP-sites [31], all of which are not conserved in DAF-16 (Supplementary Figure S1). Together, our observation of *in vivo* phosphorylation of DAF-16 at Ser326, conservation of phosphorylated motifs between DAF-16 and FOXO, and phosphorylation data on human FOXO1 [30] raised the possibility that a DYRK1A ortholog modulates DAF-16 activity in *C. elegans*.

### Loss of *mbk-1* shortens lifespan of long-lived *C. elegans* mutants

**Figure 2:**
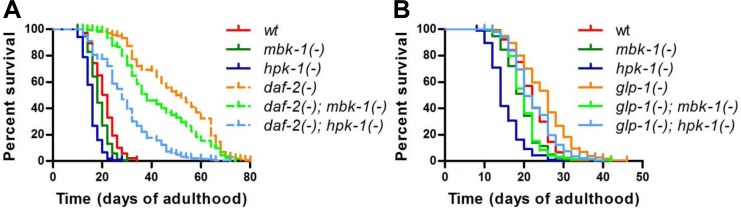
Loss of *mbk-1* decreases lifespan of long-lived *daf-2* and *glp-1* mutant *C. elegans* The effect of loss of function mutations in *mbk-1* and *hpk-1*, *mbk-1(pk1389)* and *hpk-1(pk1393),* respectively, on lifespan relative to *mbk-1(+)* and *hpk-1(+)* animals was examined in different genetic backgrounds. (**A**) *daf-2(-)* [*daf-2(e1370)*] and corresponding *daf-2(+)* animals were grown continuously at 20°C. (**B**) *glp-1(-)* [*glp-1(e2144ts)*] and corresponding *glp-1(+)* animals were grown at 25°C for the first 24 h of postembryonic development to eliminate germ cells in *glp-1(-)* strains, and subsequently, were cultured at 20°C for the remainder of the experiment. See Table 1 for statistical analysis.

To address the question whether the DYRK1A ortholog MBK-1 plays a role in *C. elegans* lifespan regulation, we introduced a predicted null mutation, *mbk-1(pk1389)* (representing 1.8-kb deletion that spans the first intron to the sixth exon and disrupts majority of the kinase domain) [26] into the long-lived *daf-2(e1370)* and *glp-1(e2144ts)* backgrounds (hereafter referred to as *mbk-1(-)*, *daf-2(-)* and *glp-1(-)*, respectively) and compared the lifespans of *mbk-1(-)* worms to that of the corresponding *mbk-1(+)* animals (Figure 2). The lifespan effects of a predicted null mutation in another DYRK-family member, *hpk-1(pk1393)* (1.5-kb deletion that disrupts the respective kinase domain) [26] were examined in parallel. *Mbk-1(-)* animals were smaller and shorter-lived than their *mbk-1(+)* counterparts in all genetic backgrounds tested, although to different extents. While *mbk-1* mutation decreased *glp-1(-)*-lifespan almost back to wild-type level, the reduction of lifespan in *daf-2(-)* and especially in wild-type animals was more modest (Figure 2, Table 1). On the other hand, *hpk-1(-)* animals appeared less healthy and were, as reported previously, substantially shorter-lived than wild-type worms [34]. Also in agreement with an earlier study [28], *hpk-1* loss strongly reduced lifespan of *daf-2(-)* worms, as well as their speed of development and viability of progeny. Interestingly, in the *glp-1(-)* background, *hpk-1(-)* mutation appeared to cause a more moderate decrease in longevity than in the other backgrounds (Figure 2, Table 1). Taken together, our lifespan analyses suggested MBK-1 as a novel factor required for full longevity of *daf-2-* and *glp-1-*deficient *C. elegans* and confirm the previously described role of *hpk-1* in maintaining normal lifespan [34].

**Table 1:** Lifespan data Related to Figure 2

Experiment	Strain	Mean survival	SEM	Deaths	Total worm number	Relative to control		Relative to wt	
						% Lifespan change	p-Value	% Lifespan change	p-Value
**# 1**	wt	20.56	0.40	151	180	N/A	N/A	N/A	N/A
*daf-2* set, graphed in Fig 2A	*mbk-1(-)*	18.40	0.27	180	200	−10.51	<0.0001	−10.51	<0.0001
	*hpk-1(-)*	15.55	0.25	174	200	−24.37	<0.0001	−24.37	<0.0001
	*daf-2(-)*	49.40	1.20	173	200	N/A	N/A	140.27	<0.0001
	*daf-2(-); mbk-1(-)*	42.47	1.06	207	210	−14.03	<0.0001	106.57	<0.0001
	*daf-2(-); hpk-1(-)*	29.09	0.83	196	200	−41.11	<0.0001	41.49	<0.0001
**# 1**	wt	21.50	0.41	123	200	N/A	N/A	N/A	N/A
*glp-1* set	*mbk-1(-)*	19.21	0.28	200	200	−10.65	<0.0001	−10.65	<0.0001
	*hpk-1(-)*	15.37	0.28	196	200	−28.51	<0.0001	−28.51	<0.0001
	*glp-1(-)*	26.39	0.54	154	200	N/A	N/A	22.74	<0.0001
	*glp-1(-); mbk-1(-)*	20.37	0.36	166	200	−22.81	<0.0001	−5.26	0.0008
	*glp-1(-); hpk-1(-)*	22.88	0.36	190	200	−13.30	<0.0001	6.42	0.3459
**# 2**	wt	16.94	0.22	136	200	N/A	N/A	N/A	N/A
	*mbk-1(-)*	17.80	0.20	220	220	5.08	0.0888	5.08	0.0888
	*hpk-1(-)*	12.72	0.14	176	200	−24.91	<0.0001	−24.91	<0.0001
	*glp-1(-)*	24.14	0.62	187	240	N/A	N/A	42.50	<0.0001
	*glp-1(-); mbk-1(-)*	20.81	0.18	360	400	−13.79	<0.0001	22.85	<0.0001
	*glp-1(-); hpk-1(-)*	20.32	0.43	167	180	−15.82	<0.0001	19.95	<0.0001
**# 3**	wt	20.76	0.35	199	220	N/A	N/A	N/A	N/A
	*mbk-1(-)*	19.63	0.31	239	250	−5.44	0.0028	−5.44	0.0028
	*hpk-1(-)*	13.68	0.23	165	200	−34.10	<0.0001	−34.10	<0.0001
	*glp-1(-)*	22.23	0.46	150	200	N/A	N/A	7.08	0.0025
	*glp-1(-); mbk-1(-)*	18.39	0.15	234	300	−17.27	<0.0001	−11.42	0.0028
	*glp-1(-); hpk-1(-)*	20.27	0.44	184	200	−8.82	0.0013	−2.36	<0.0001
**# 4**	wt	19.12	0.42	170	200	N/A	N/A	N/A	N/A
	*glp-1(-)*	24.72	0.70	181	200	N/A	N/A	29.29	<0.0001
	*glp-1(-); mbk-1(-)*	17.83	0.46	129	150	−27.87	<0.0001	−6.75	0.0192
	*glp-1(-); hpk-1(-)*	19.45	0.64	99	150	−21.32	<0.0001	1.73	0.6228
**composite**	wt	19.83	0.22	458	620	N/A	N/A	N/A	N/A
combined *glp-1* sets from # 1/2/3, graphed in Fig 2B	*mbk-1(-)*	18.89	0.16	659	670	−4.74	0.0004	−4.74	<0.0001
	*hpk-1(-)*	13.98	0.14	537	600	−29.50	<0.0001	−29.50	<0.0001
	*glp-1(-)*	24.26	0.33	491	640	N/A	N/A	22.34	<0.0001
	*glp-1(-); mbk-1(-)*	19.97	0.13	760	900	−17.68	<0.0001	0.71	0.0181
	*glp-1(-); hpk-1(-)*	21.21	0.24	541	580	−12.57	<0.0001	6.96	0.0049

The effect of the *mbk-1(pk1389)* and *hpk-1(pk1393)* loss of function mutations on lifespan relative to *mbk-1(+)* and *hpk-1(+)* animals was examined in different genetic backgrounds. % change in lifespan and p-Values from Mantel-Cox-tests were calculated relative to *mbk-1(+)* and *hpk-1(+)* control animals of the same genetic background (*wt, daf-2(-) or glp-1(-)*), and relative to the wild-type strain N2E. Experiment #1, *daf-2* set was plotted in Figure 2A, combined data for the *glp-1* sets. Experiments 1, 2 and 3 were plotted in Figure 2B. Lifespan increases observed for *glp-1* relative to wild-type are consistent with experiments in the literature [51, 63, 64].

### Loss of *mbk-1* reduces DAF-16 target gene expression

**Figure 3:**
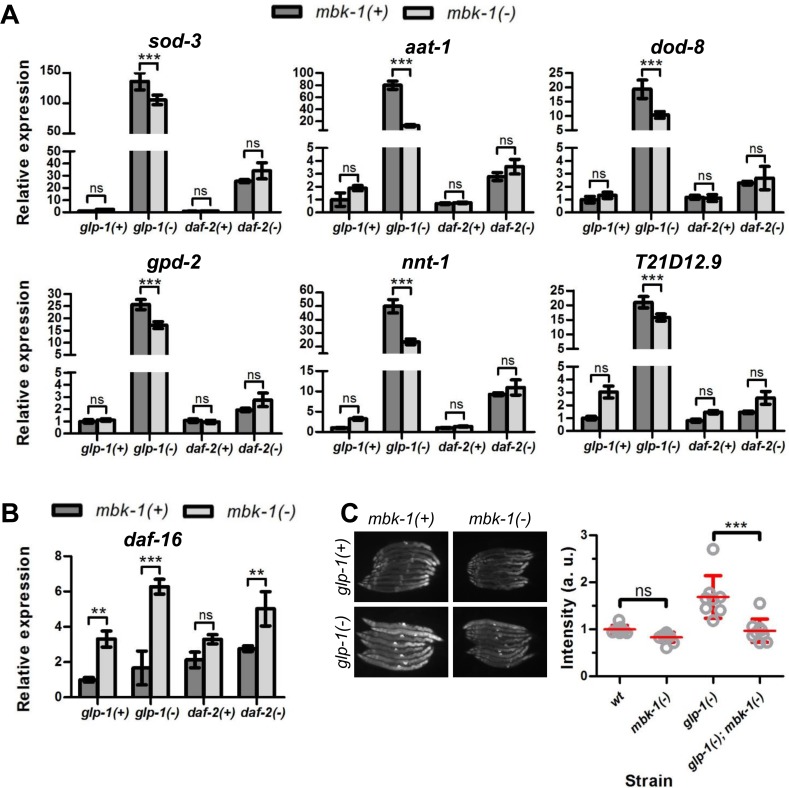
Effect of *C. elegans mbk-1* on DAF-16 target gene expression (**A**) Loss of *mbk-1* decreases expression of a panel of DAF-16 target genes in *glp-1(-)* [*glp-1(e2144ts)*], but not in wild-type or *daf-2(-)* [*daf-2(e1370)*] animals as determined by qPCR (representative experiment shown, n=2). Error bars indicate standard deviations of three technical replicates. Statistical significance of expression level differences was determined by two-way ANOVA with Bonferroni post tests. (**B**) Loss of *mbk-1* does not decrease *daf-16* mRNA levels as determined by qPCR (representative experiment shown, n=2; error bars and statistical analysis as in panel **A**). (**C**) Loss of *mbk-1* decreases *Psod-3::gfp*-expression in *glp-1(-)*, and –to a lesser extent- in wild-type background (representative experiment shown, n=3). Error bars indicate standard deviations. Statistical significance of fluorescence intensity differences was determined by two-way ANOVA with Bonferroni post tests. All experiments in (**A**)-(**C**) were performed on day-2 adult worms. Images in (**C**) were taken at 100x magnification.

To investigate whether the reduction of *glp-1(-)* and *daf-2(-)* longevity upon *mbk-1* inactivation is due to DAF-16-inhibition, we used qPCR to measure the mRNA levels of eight genes that previously have been reported to be upregulated by DAF-16 upon germline ablation and/or *daf-2* mutation [16, 35, 36], in wild-type, *glp-1(-)* and *daf-2(-)* worms. Transcripts of six genes, *sod-3*, *aat-1*, *dod-8*, *gpd-2*, *nnt-1* and *T21D12.9,* were strongly induced in germline-deficient *mbk-1(+)* worms but consistently lowered when *mbk-1* was inactivated in these animals (Figure 3A). In contrast, *F52H3.5* and *K07B1.4* expression levels were not significantly affected by *mbk-1* loss (data not shown). In *daf-2(-)* animals, expression of all genes analyzed was also elevated relative to wild-type worms, but not significantly reduced in the absence of *mbk-1* (Figure 3A). Similarly, in wild-type background, *mbk-1* loss also did not suppress DAF-16 target genes (Figure 3A). Of note, mRNA levels of *daf-16* itself were not decreased, but rather, increased in *mbk-1(-)* animals in the three genetic backgrounds examined (Figure 3B).

The role of *mbk-1* in modulating expression of the well-characterized *daf-16* regulated gene *sod-3* [37, 38] was also analyzed using a *Psod-3::gfp* reporter-construct [36]. In agreement with the qPCR results, *mbk-1(pk1389)* consistently lowered *Psod-3::gfp* fluorescence in the *glp-1(-)* background and also in wild-type, although to a lesser extent and not statistically significantly (Figure 3C, Supplementary Table 3). For the lifespan-shortening *hpk-1(-)* allele, there seemed to be trend towards decreased *Psod-3::gfp* expression in *glp-1(-)*; *hpk-1(-)* worms, while the opposite was observed in *hpk-1(-)* single mutant worms (Supplementary Figure S2A and Supplementary Table 4). When *mbk-2*, the third DYRK-family member in *C. elegans,* was depleted by RNAi (null mutations in *mbk-2* cause embryonic lethality [26]), we consistently observed elevated *Psod-3::gfp* levels in *glp-1(-)* animals relative to control RNAi-treated animals, and similar trends were seen in wild-type worms (Supplementary Figure S2B, Supplementary Table 5). Of note, RNAi-depletion of *mbk-2* in wild-type worms also caused the prominent *mbk-2* phenotype of almost 100% dead eggs [26, 39]. Taken together, our qPCR and reporter gene analyses indicate that *mbk-1* loss prevents full induction of a subset of DAF-16 target genes in *glp-1(-)*-animals but does not attenuate expression of the same DAF-16 targets in the wild-type or *daf-2(-)* background.

### Loss of *mbk-1* does not block DAF-16 nuclear accumulation in germline-deficient *C. elegans*

**Figure 4:**
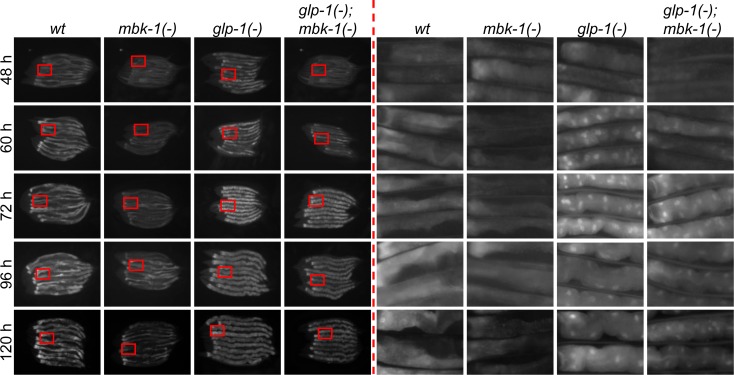
Loss of *mbk-1* does not affect DAF-16 subcellular localization in germline-deficient *C. elegans* The effect of the *mbk-1* loss of function mutation *mbk-1(pk1389)* on subcellular localization of an intestine-specific GFP::DAF-16 protein (encoded by transgene *muIs145[Pges-1::gfp::daf-16]*) was determined at the times indicated in wild-type and germline-deficient *glp-1(-)* [*glp-1(e2144ts)*] animals. Images on the left were taken at 128x (48 h), 100x (60-96h) or 80x (120 h) magnification, images on the right are 6.5x magnifications of the areas boxed in red.

To examine whether MBK-1 affects DAF-16 target gene expression in *glp-1(-)* worms by altering DAF-16 subcellular localization, we analyzed nuclear accumulation of GFP::DAF-16 expressed specifically in the intestine in the presence and absence of the *mbk-1(-)* mutation in wild-type and *glp-1(-)* worms by fluorescence microscopy. In all *glp-1(+)* animals, the intestine-specific (*ges-1* promoter-driven) GFP::DAF-16 protein was predominantly cytoplasmic at all time points analyzed (48 h - 120 h post plating of L1 larvae, i.e. from the L4 stage until day 3 of adulthood, Figure 4). In agreement with a previous report [18], nuclear accumulation of GFP::DAF-16 in *glp-1(-)* single-mutant animals began in early adulthood and was essentially complete 60 h after plating of L1 larvae. On the other hand, in *glp-1(-); mbk-1(-)* double-mutant animals, nuclear accumulation of GFP::DAF-16 was slightly delayed and completed only 72 h after plating. However, this delay in GFP::DAF-16 nuclear accumulation appeared to parallel the general slight delay in postembryonic development that is conferred by *mbk-1* loss (data not shown). Since blocking phosphorylation of the DAF-16 ortholog FOXO1 at the site regulated by the MBK-1 ortholog DYRK1A in human cells has been reported to further increase FOXO1 nuclear accumulation under conditions of low IGF-1 signaling [30], we also examined GFP::DAF-16 localization in *daf-2(-)* animals and found that it was also not altered by the *mbk-1* mutation (Supplementary Figure S3). Therefore, we conclude that in the conditions tested, MBK-1 does not regulate DAF-16 subcellular localization and instead, may control its transcriptional activity through other mechanisms.

## DISCUSSION

In this study, we report the first evidence for the DYRK1A ortholog MBK-1 contributing to lifespan extension in response to germline ablation and decreased insulin-like signaling in *C. elegans*. Moreover, our data indicate that MBK-1 exerts at least parts of its lifespan-modulatory function in germline-less/*glp-1(-)* worms by maximizing the activity of the FOXO-transcription factor DAF-16. On the other hand, in insulin receptor/*daf-2(-)* animals, *mbk-1* inactivation did not reduce the expression of a subset of DAF-16 target genes. Thus, an MBK-1/DAF-16 signaling axis may act specifically in the context of germline deficiency to promote longevity, while contribution of MBK-1 to *daf-2(-)* longevity may be mediated by other factors.

Our study was initiated by the observation that DAF-16 Ser326 is phosphorylated in wild-type worms. Intriguingly, the DAF-16 ortholog FOXO1 has been reported to be inhibited by phosphorylation at the corresponding site, Ser329 in unstimulated and IGF-1 stimulated cultured cells [30]. Moreover, FOXO1-Ser329 has been identified as a major *in vitro* phosphorylation site of the mammalian kinase DYRK1A [30]. Thus, its *C. elegans* ortholog MBK-1 appeared to be a good candidate negative regulator of *daf-16* dependent longevity pathways. However, our results in wild-type and *daf-2(-)* worms, which parallel IGF-1 treated and untreated cells examined previously [30], indicated that MBK-1 does not influence DAF-16 transcriptional activity and subcellular localization under these conditions. We note that our analysis focused on DAF-16 target genes reported previously to be induced in response to lifespan-extending genetic mutations [16, 35, 36]. Moreover, the *daf-16* locus, through the use of different promoters and transcriptional start sites and through alternative splicing, gives rise to several isoforms with partially different expression patterns and target gene profiles [40, 41]. Longevity of *daf-2(-)* and also of *glp-1(-)* worms (cf. below) appears to be predominantly mediated by isoform a (referred to as isoform c in this study), while contributions from DAF-16f are controversial [40, 41]. Thus, our data cannot rule out the possibility that MBK-1 in wild-type and *daf-2(-)* worms regulates DAF-16 isoforms and target genes that were not examined by us. Yet, our *C. elegans* results in combination with currently available mammalian cell data, are also consistent with the notion that DYRK1A-regulation of FOXO transcription factors is not conserved across species and/or may even be specific to FOXO1. Indeed, potential DYRK1A-phosphorylation of FOXO3 and FOXO4, which share the DYRK1A-site but not all of their organismal functions with FOXO1 [1], has not been investigated yet.

On the other hand, our observation that *mbk-1* loss reduces longevity and DAF-16 target gene expression in *glp-1* deficient *C. elegans* is consistent with the model that MBK-1 is a positive regulator of DAF-16 activity and lifespan extension. Moreover, conservation of the DYRK1A-site between FOXO1 and DAF-16 supports the hypothesis that DAF-16 is a substrate of MBK-1. However, such a model in *C. elegans* substantially differs from the model suggested by previous work in mammalian cells [30], which implies that DYRK1A is an inhibitor of FOXO1. This discrepancy raises the possibility that a potential MBK-1/DAF-16 signaling axis in *C. elegans* does not parallel the apparent DYRK1A/FOXO1 kinase-substrate relationship in mammalian cells in all details. Interestingly, recent reports already suggested that regulatory pathways can differ between *C. elegans* and mammals although they engage orthologous factors. For example, the deubiquitylase MATH-33 recently has been reported to stabilize/activate DAF-16 by antagonizing polyubiquitylation, while its mammalian counterpart USP7/HAUSP inhibits FOXO1 and FOXO4 by decreasing their nuclear localization and transcriptional activity, respectively, by removing monoubiquitin moieties [42–44]. Moreover, MBK-1 itself may function differently from its human orthologs DYRK1A and DYRK1B, at least in certain contexts. Specifically, MBK-1 promotes transcriptional activity of HIF-1, *C. elegans’* only hypoxia-inducible factor α subunit [45], independently of the HIF-1 destabilizing E3-ligase VHL-1, thereby contributing to *Pseudomonas aeruginosa* resistance [27]. In contrast, in glioma stem cells, one of the human HIF−1 homologs, HIF-2α/EPAS1, is inhibited by DYRK1A/B in a VHL-dependent manner [46]. It will be interesting to examine the role of DAF-16 Ser326 phosphorylation and of other Ser326 candidate kinases, such as the MBK-1 relative MBK-2 [26] and the NLK-ortholog LIT-1 [47], on *C. elegans* lifespan and on global DAF-16 target gene expression. Such studies will, together with biochemical studies on DAF-16 and putative Ser326 kinases, further clarify the mechanistic links between longevity, DAF-16 Ser326-phosphorylation, and MBK-1 in *C.elegans.*

Although *mbk-1* loss in our study only partially suppressed DAF-16 target gene expression in *glp-1(-)* worms, it completely prevented lifespan extension in these animals. Accordingly, MBK-1 may regulate other germline-longevity promoting factors in addition to DAF-16, for example SKN-1, PHA-4, DAF-12 or NHR-80 [17]. In contrast, in *daf-2(-)* animals, *mbk-1* loss shortened lifespan without significantly attenuating the induction of the DAF-16 target genes analyzed. Therefore, as discussed above, *mbk-1* may contribute to *daf-2(-)* longevity by engaging factors other than DAF-16, for example SKN-1 or HSF-1 [2]. Interestingly, similar to MBK-1 in this study, the transcription elongation factor TCER-1 and the adaptor protein KRI-1, have been reported previously to modulate DAF-16 activity only in *glp-1(-)*, but not in *daf-2(-)* animals [21, 36]. The concept that *daf-2* and *glp-1* mutations influence DAF-16 activity through different signaling mediators is further supported by a recent study that provided evidence for both, *daf-2(-)* and *glp-1(-)* longevity being primarily dependent on the same DAF-16 isoform, DAF-16a [40].

*Mbk-1* has been implicated in several longevity-relevant processes, including pathogen resistance, H_2_S resistance and HIF-1 activation [27]. For *daf-16* a role in antibacterial immunity has also been described, which involves protection against strains that kill *C. elegans* slowly by gut colonization [48, 49]. *Mbk-1*, on the other hand, counteracts fast-killing of worms by the HCN-producing *Pseudomonas aeruginosa* strain PAO1 [27]. Whether *daf-16* contributes to the *mbk-1* mediated defense mechanism or vice versa, has not been examined. Since *mbk-1* mediated resistance against the PAO1 strain likely reflects a function of MBK-1 in increasing HCN-tolerance, MBK-1 may also protect *C. elegans* from other toxic compounds with similar modes of action to HCN, such as H_2_S [27, 50]. Interestingly, elevated levels of endogenous H_2_S have been observed in germline-deficient worms and have been reported to be required for their longevity [51, 52]. Thus, it is tempting to speculate that MBK-1 enables germline-deficient worms to tolerate higher endogenous H_2_S levels. However, the described mechanism for MBK-1 mediated resistance against HCN, and by extension H_2_S, further involves the transcription factor HIF-1 [27, 53], which is not required for longevity of both, *glp-1* and *daf-2* mutant *C. elegans* [54, 55]. It will be interesting to examine the role of MBK-1 in protection from H_2_S in the future.

In summary, the data reported here establish an unanticipated positive role for the conserved protein kinase MBK-1 in the longevity of *daf-2* and germline-deficient *C. elegans* and point to regulatory connections between MBK-1 and DAF-16 that are different form the DYRK1A-FOXO1 axis in mammalian cells.

## MATERIALS AND METHODS

### *C. elegans* strains and culture

Strains used in this study are listed in Supplementary Table 1. Worms were cultured on NG agar plates seeded with *E. coli* OP50 according to standard protocols. To eliminate germ cells in worms carrying the *glp-1(e2144ts)* allele, these animals and corresponding *glp-1(+)* control animals were incubated at 25°C for the first 24 h of postembryonic development at then shifted to 20°C. *daf-2(e1370)* worms and corresponding *daf-2(+)* control worms were continuously cultured at 20°C.

### Bioinformatics analysis

Protein sequence alignments of human FOXO1/3/4/6 (UniProt accession numbers Q12778, O43524, P98177, A8MYZ6, last retrieval on 05/01/2016) and DAF-16 isoform c/a1 (O16850-3) were performed using the ClustalΩ program at www.uniprot.org. All *daf-16* transgenes used in this study and numbering in DAF-16 sequences correspond to isoform c/a1.

### GFP::DAF-16 immunoprecipitation

For mass spectrometry experiments, worms expressing GFP or GFP-tagged DAF-16 in the intestine (*zcIs18[Pges-1::gfp(cyt)* or *muIs194[Pges-1::ha::gfp::daf-16 + Podr-1::rfp*]) were synchronized by hypochlorite treatment and grown at a density of 4,000 worms/10 cm plate until day 1 of adulthood. Approx. 200,000 worms were grown in three batches, harvested, flash frozen and combined upon lysis by bead-beating (BioSpec Products, Bartlesville, OK, USA) with 0.7 mm Zirconia beads in 2 pellet volumes of lysis buffer (modified from [66]: 50 mM HEPES pH 7.4, 100 mM NaCl, 1 mM EGTA, 10% glycerol) containing 2x protease and phosphatase inhibitors (2 mM PMSF, complete and PhosSTOP^TM^ tablets, Roche Diagnostics, Rotkreuz, Switzerland). Then, detergents were added to final concentrations of 1% Triton X-100, 1% Sodium Deoxycholate and 0.1% SDS and lysates were incubated under rotation at 4°C for 15 min. Lysates were cleared by 4 rounds of centrifugation at 14,000 rpm, 4°C, 15 min and incubation with unconjugated agarose beads. GFP/GFP::DAF-16 was immunoprecipitated from 30 mg of total protein lysate (20 mg/ml) using an anti-GFP nanobody coupled to agarose beads (GFPtrap, ChromoTek, Planegg-Martinsried, Germany). Beads were washed four times with lysis buffer with detergents and 1x inhibitors, once with high salt buffer (10 mM Tris, pH 7.4, 500 mM NaCl) and once with low salt buffer (10 mM Tris, pH 7.4, 100 mM NaCl). For mass spectrometry analyses, beads were eluted with 2% SDS, 50 mM Tris, pH 6.8, 5% v/v beta-Mercaptoethanol.

### Protein digestion

Eluates were diluted to 8 M urea - 100 mM Tris(hydroxyethylamine) pH 8.4 for denaturation and reduction of proteins with 5 mM Tris(2-carboxyethyl) phosphine for 30 min. Cysteine residues were acetylated with 10 mM iodoacetamide for 15 min in the dark. The sample was diluted to 2 M urea with 100 mM Tris(hydroxyethylamine) pH 8.5. Trypsin (0.5 µg) and CaCl_2_ (1 mM) were added for a 4 hour digestion at 37°C. The peptide sample was acidified to 5% formic acid and spun at 18,000 x g and loaded directly onto a MudPIT column.

### MudPIT analysis

Capillary columns were prepared in-house for LC-MS/MS analysis from particle slurries in methanol. An analytical RPLC column was generated by pulling a 100 µm ID/360 µm OD capillary (Polymicro Technologies, Inc, Phoenix, AZ) to a 5 µm ID tip. Reverse phase particles (Jupiter C18, 4 µm dia., 90 Å pores, Phenomenex, Torrance, CA) were packed directly into the pulled column at 800 psi until 15 cm long. The column was further packed, washed, and equilibrated at 100 bar with buffer B followed by buffer A. MudPIT and analytical columns were assembled using a zero-dead volume union (Upchurch Scientific, Oak Harbor, WA). LC-MS/MS analysis was performed using an Agilent 1200 HPLC pump and Thermo LTQ-Orbitrap XL using an in-house built electrospray stage. Electrospray was performed directly from the analytical column by applying the ESI voltage at a tee (150 µm ID, Upchurch Scientific) directly downstream of a 1:1000 split flow used to reduce the flow rate to 250 nL/min through the columns. 3-step MudPIT [56] was performed where each step corresponds to 0, 25, and 100% buffer C being run for 5 min at the beginning of a 2 hr gradient. The repetitive 2 hr gradients were from 100% buffer A to 60% buffer B over 70 min, up to 100% B over 20 min, held at 100% B for 10 min, then back to 100% A for a 10 min column re-equilibration. Buffer A was 5% acetonitrile 0.1% formic acid, B was 80% acetonitrile 0.1% formic acid, and C was 500 mM ammonium acetate. Electrospray directly from the LC column was done at 2.5 kV with an inlet capillary temperature of 250°C. Precursor scanning in the Orbitrap XL was performed from 400 - 2000 m/z with the following settings: 5 × 10^5^ target ions, 50 ms maximum ion injection time, and 1 microscan. Data-dependent acquisition of MS/MS spectra with the LTQ on the Orbitrap XL were performed with the following settings: MS/MS on the 8 most intense ions per precursor scan, 30K automatic gain control target ions, 100 ms maximum injection time, and 1 microscan. Dynamic exclusion settings used were as follows: repeat count: 1; repeat duration: 30 sec; exclusion list size: 500; and exclusion duration: 60 sec. Protein and phosphopeptide identification and phosphorylation analysis were performed using Integrated Proteomics Pipeline (IP2, www.integratedproteomics.com/). Tandem mass spectra were extracted to MS2 files from raw files using RawExtract 1.9.9 [57] and searched against a non-redundant UniProt human database with reversed sequences using ProLuCID [58]. The search space included all fully- and half-tryptic peptide candidates. Carbamidomethylation (+57.02146) of cysteine was considered as a static modification; phosphorylation (+79.9663) on serine, threonine, and tyrosine were considered as variable modifications. Peptide candidates were filtered to 0.1% FDR using DTASelect [59].

### Lifespan analysis

To obtain synchronized populations, gravid adults were treated with hypochlorite and eggs were allowed to hatch in M9 buffer overnight. L1 larvae were plated on NG agar plates seeded with *E. coli* strain OP50. At the late L4 stage, and every 10 days thereafter, worms were transferred to fresh OP50-seeded NG agar plates containing 20 µM 5-fluoro-2′-deoxyuridine (FUDR) to prevent development of progeny and desiccation, respectively. Animals were maintained at a density of 40 worms/6 cm plate and scored for survival every other day starting on day 8 of adulthood. Worms were considered dead if they did not respond to gentle touching with a worm pick. Animals that showed a protruding vulva, or had ruptured, died from internal progeny hatching (bagging) or escaped from the plate, were censored. Kaplan-Meier survival analysis was performed using, GraphPad Prism 5 (GraphPad Software, La Jolla, CA, USA).

### RNAi experiments

All RNAi clones were from the Ahringer library (Source BioScience, Nottingham, UK) and verified by sequencing. The empty vector L4440 served as control. Experiments were performed as described previously [51]. RNAi treatment was initiated in the L1 stage unless otherwise noted.

### Fluorescence imaging

Worms expressing *muIs84[Psod-3::gfp]* [38] or *muIs145[Pges-1::gfp::daf-16+Podr-1::rfp]* (integrated version of *muEx268* [38]) were synchronized by timed egg laying for 2 h and analyzed on day 2 of adulthood (unless otherwise noted) using a fluorescence microscope equipped with a standard GFP bandpass filter (MF16, Leica Microsystems, Wetzlar, Germany). GFP signal intensity in *muIs84*-expressing animals was quantified with Cellprofiler (http://cellprofiler.org) [60].

### qPCR

RNA was extracted from 200 synchronized day 2 adults using TRIzol Reagent (Life Technologies/Thermo Fisher Scientific, Waltham, MA, USA), and 0.5-2 µg total RNA were reverse-transcribed using the Protoscript First Strand Synthesis kit (New England Biolabs, Ipswich, MA, USA). qPCR was performed on an AbiPrism 7300 instrument (Applied Biosystems®/Thermo Fisher Scientific, Waltham, MA, USA) with SYBR® Green (Power SYBR® Green Master Mix, Applied Biosystems®/Thermo Fisher Scientific, Waltham, MA, USA). Data were analyzed by the ΔΔCt method and target gene expression levels were normalized to the geometric mean of *cdc-42*, *tba-1* and *Y45F10D.4* [61, 62]. Primers for qPCR analysis of DAF-16 target genes have been published previously [36].

## SUPPLEMENTARY MATERIAL


