# Supplementary material for: The protein kinase MBK-1 contributes to lifespan extension in daf-2 mutant and germline-deficient Caenorhabditis elegans
Source: Aging (Albany NY). 2017 May 25;9(5):1414–26. doi: 10.18632/aging.101244 (PMC5472741; doi:10.18632/aging.101244)
Supplement: Supplementary file 1 [file aging-09-1414-s001.pdf]

SUPPLEMENTARY MATERIAL

Supplementary Table 1. List of strains used in this study.

Strain	genotype	comment
N2E		Wild-type
CF3942	*glp-1(e2144ts) III*	*glp-1(e2144ts)* from CF1903 [21], outcrossed 12x to N2E
CF3943	*muIs84[Psod-3::gfp]*	*muIs84* from CF1553 [38] outcrossed 12x
CF3949	*glp-1(e2144ts) III; muIs84[Psod-3::gfp]*	
CF4339	*daf-2;(e1370) III; muIs84[Psod-3::gfp]*	
CF4054	*daf-16(mu86) I*	*daf-16(mu86)* from CF1037 [18], outcrossed 12x to N2E
CF4087	*daf-2(e1370) III*	*daf-16(mu86)* from CF1041 [18], outcrossed 12x
CF4096	*daf-16(mu86) I; muIs194[Pges-1::ha::gfp::daf-16 + Podr-1::rfp]*	*muIs194* from CF3628: *daf-16(mu86) I; muIs194*
CF4117	*zcIs18[Pges-1::gfp(cyt)]*	Strain SJ4144 (Ron lab/CGC) outcrossed 6x
CF4164	*mbk-1(pk1389) X*	*mbk-1(pk1389)* from EK228 [26] (Kandel lab /CGC) outcrossed 6x
CF4165	*glp-1(e2144ts) III; mbk-1(pk1389) X*	
CF4166	*daf-2(e1370) III; mbk-1(pk1389) X*	
CF4167	*daf-16(mu86) I; muIs145[Pges-1::gfp::daf-16 + Podr-1::rfp]*	*muIs145* is the integrated version of muEx268 [38]
CF4168	*daf-16(mu86) I; glp-1(e2144ts) III; muIs145[Pges-1::gfp::daf-16 + Podr-1::rfp]*	
CF4169	*daf-16(mu86) I;daf-2(e1370) III; muIs145[Pges-1::gfp::daf-16 + Podr-1::rfp]*	
HMT029	*daf-16(mu86) I; mbk-1(pk1389) X; muIs145[Pges-1::gfp::daf-16 + Podr-1::rfp]*	
HMT030	*daf-16(mu86) I; glp-1(e2144ts) III; mbk-1(pk1389) X; muIs145[Pges-1::gfp::daf-16 + Podr-1::rfp]*	
HMT031	*daf-16(mu86) I; daf-2(e1370) III; mbk-1(pk1389) X; muIs145[Pges-1::gfp::daf-16 + Podr-1::rfp]*	
CF4173	*hpk-1(pk1393) X*	*hpk-1(pk1393)* from EK273 [26] (Kandel lab/CGC) outcrossed 6x
CF4185	*glp-1(e2144ts) III; hpk-1(pk1393) X*	
HMT001	*daf-2(e1370) III; hpk-1(pk1393) X*	Very low progeny, reported to be synthetic lethal [34]
CF4183	*hpk-1(pk1393) X; muIs84[Psod-3::gfp]*	
HMT002	*glp-1(e2144ts) III; hpk-1(pk1393) X; muIs84[Psod-3::gfp]*	
CF4184	*mbk-1(pk1389) X; muIs84[Psod-3::gfp]*	
HMT003	*glp-1(e2144ts) III; mbk-1(pk1389) X; muIs84[Psod-3::gfp]*	
HMT004	*daf-2(e1370) III; mbk-1(pk1389) X; muIs84[Psod-3::gfp]*	

Supplementary Table 2. List of qPCR primers used in this study.

primer name	primer sequence 5'-->3'
cdc-42_RT_F	TCA GCG TTG ACG CAG AAG
cdc-42_RT_R	CAT GGA GAC AAG GAA GAC GTT
tba-1_RT_F	TCC ACT GAT CTC TGC TGA CAA
tba-1_RT_R	TGG ATC GCA CTT CAC CAT T
Y45F10D.4_RT_F	AAG CGT CGG AAC AGG AAT C
Y45F10D.4_RT_R	TTT TTC CGT TAT CGT CGA CTC
daf-16_RT_F	TAC GAA TGG ATG GTC CAG AA
daf-16_RT_R	TCG CAT GAA ACG AGA ATGA A
sod-3_RT_F	AAA GGA GCT GAT GGA CAC TAT TAA GC
sod-3_RT_R	AAG TTA TCC AGG GAA CCG AAG TC
aat-1_RT_F	CCC AAA ACG AAA CCT TCC ACT CGC
aat-1_RT_R	TGA AAT TGC TGT GTA GAG AGC CAC
dod-8_RT_F	ACA GGA TGT CTT CAA AAG GAA TAT GG
dod-8_RT_R	TTG CTG GGG TGA TAG CTT GG
gpd-2_RT_F	AAG GCC AAC GCT CAC TTG AA
gpd-2_RT_R	GGT TGA CTC CGA CGA CGA AC
F52H3.5_RT_F	GAA GTT TAC AAA AGC ACT CGA AG
F52H3.5_RT_R	GGT TTA TTT TGA AGT CGG TAT GC
K07B1.4_RT_F	GGT CTT CTT CCA TTC AGA AAA CC
K07B1.4_RT_R	TGT ATG TCT GAT GAA GTG TGT CG
nnt-1_RT_F	CAG TAG AAA CTG CTG ACA TGC TTC
nnt-1_RT_R	GAG CGA TGG GAT ATT GTG CCT GAG
T21D12.9_RT_F	CAT CTA AAT CTA TCA ACT AAT AGA G
T21D12.9_RT_R	GTA GGA CAG GTC CAA AAC TTC CAA G

Supplementary Table 3. Effect of *mbk-1* loss on *Psod-3::gfp*-expression in wild-type and germline-deficient *C. elegans*. Related to Figure 3C.

Experiment	Strain	Worm number	Fold-change expression relative to wt			Fold-change expression relative to *glp-1(-)*			P-value
			Mean	SD	SEM	Mean	SD	SEM	
#1	wt	24	1.00	0.15	0.03	0.38	0.06	0.01	
	mbk-1(-)	24	0.91	0.16	0.03	0.34	0.06	0.01	>0.05
	glp-1(-)	22	2.65	1.07	0.23	1.00	0.40	0.09	
	glp-1(-); mbk-1(-)	9	0.99	0.22	0.07	0.37	0.08	0.03	<0.001
#2	wt	24	1.00	0.20	0.04	0.47	0.09	0.02	
	mbk-1(-)	20	0.82	0.32	0.07	0.39	0.15	0.03	>0.05
	glp-1(-)	15	2.12	0.71	0.18	1.00	0.34	0.09	
	glp-1(-); mbk-1(-)	10	0.87	0.22	0.07	0.41	0.10	0.03	<0.001
#3	wt	9	1.00	0.08	0.03	0.59	0.05	0.02	
	mbk-1(-)	9	0.83	0.10	0.03	0.49	0.06	0.02	>0.05
	glp-1(-)	8	1.69	0.45	0.16	1.00	0.27	0.09	
	glp-1(-); mbk-1(-)	9	0.97	0.25	0.08	0.58	0.15	0.05	<0.001

The effect of the *mbk-1* loss of function mutation *mbk-1(pk1389)* on the expression of a *Psod-3::gfp* reporter gene *(muIs84)* relative to *mbk-1(+)* animals was examined in wild-type and germline-less, *glp-1(-)* [*glp-1(e2144ts)*] worms. Fluorescence images were quantified, corrected for background, and fold-changes in reporter gene expression were calculated relative to wild-type and *glp-1(-)* animals. Statistical significance was determined by two-way ANOVA with Bonferroni post tests. Experiment #3 is shown in Figure 3C.

Supplementary Table 4. Effect of *hpk-1* loss on *Psod-3::gfp*-expression in wild-type and germline-deficient *C. elegans.* Related to Supplementary Figure S2A.

Experiment	Strain	Worm number	Fold-change expression relative to wt			Fold-change expression relative to *glp-1(-)*			P-value
			Mean	SD	SEM	Mean	SD	SEM	
#1	wt	24	1.00	0.15	0.03	0.38	0.06	0.01	
	hpk-1(-)	23	1.65	0.26	0.05	0.62	0.10	0.02	<0.01
	glp-1(-)	22	2.65	1.07	0.23	1.00	0.40	0.09	
	glp-1(-); hpk-1(-)	22	2.60	0.59	0.13	0.98	0.22	0.05	>0.05
#2	wt	24	1.00	0.20	0.04	0.47	0.09	0.02	
	hpk-1(-)	14	1.27	0.43	0.12	0.60	0.21	0.05	>0.05
	glp-1(-)	15	2.12	0.71	0.18	1.00	0.34	0.09	
	glp-1(-); hpk-1(-)	3	1.71	0.65	0.37	0.81	0.31	0.18	>0.05
#3	wt	9	1.00	0.08	0.03	0.59	0.05	0.02	
	hpk-1(-)	25	1.14	0.20	0.04	0.67	0.12	0.02	>0.05
	glp-1(-)	8	1.69	0.45	0.16	1.00	0.27	0.09	
	glp-1(-); hpk-1(-)	17	1.30	0.14	0.03	0.77	0.08	0.02	<0.001

The effect of the *hpk-1* loss of function mutation *hpk-1(pk1393)* on the expression of a *Psod-3::gfp* reporter gene *(muIs84)* relative to *hpk-1(+)* animals was examined in wild-type and germline-less, *glp-1(-)* [*glp-1(e2144ts)*] worms. Fluorescence images were quantified, corrected for background, and fold-changes in reporter gene expression were calculated relative to wild-type and *glp-1(-)* animals. Statistical significance was determined by two-way ANOVA with Bonferroni post tests. Experiment #3 is shown in Supplementary Figure S2A. Note: In Experiment #3, 3 images were taken for *hpk-1(-)* and 2 images for *glp-1; hpk-1(-)*.

Supplementary Table 5. Effect of *mbk-2* knockdown on *Psod-3::gfp*-expression in wild-type and *germline-deficient C. elegans*. Related to Supplementary Figure S2B.

			Fold-change expression relative to wt			Fold-change expression relative to *glp-1*			
**Experiment**	**Strain/RNAi**	**Worm number**	**Mean**	**SD**	**SEM**	**Mean**	**SD**	**SEM**	**P-value**
#1	wt/control	7	1.00	0.12	0.04	0.47	0.06	0.02	
	wt/*mbk-2*	10	1.35	0.27	0.08	0.64	0.13	0.04	>0.05
	glp-1(-)/control	8	2.12	0.76	0.27	1.00	0.36	0.13	
	glp-1(-)/*mbk-2*	10	2.90	0.65	0.21	1.37	0.31	0.10	<0.01
#2	wt/control	9	1.00	0.16	0.05	0.51	0.08	0.03	
	wt/*mbk-2*	9	1.78	0.22	0.07	0.91	0.11	0.04	<0.001
	glp-1(-)/control	10	1.96	0.51	0.16	1.00	0.26	0.08	
	glp-1/*mbk-2*	10	5.10	0.57	0.18	2.60	0.29	0.09	<0.001
#3	wt/control	10	1.00	0.09	0.03	0.58	0.05	0.02	
	wt/*mbk-2*	10	1.22	0.19	0.06	0.71	0.11	0.04	>0.05
	glp-1(-)/control	10	1.72	0.30	0.10	1.00	0.18	0.06	
	glp-1(-)/*mbk-2*	10	2.30	0.69	0.22	1.34	0.40	0.13	<0.01
#4	wt/control	16	1.00	0.06	0.01	0.60	0.03	0.01	
	wt/*mbk-2*	11	1.06	0.07	0.02	0.64	0.04	0.01	>0.05
	glp-1(-)/control	20	1.65	0.29	0.06	1.00	0.17	0.04	
	glp-1(-)/*mbk-2*	8	2.02	0.53	0.19	1.22	0.32	0.11	<0.01
#5	wt/control	10	1.00	0.19	0.06	0.42	0.08	0.03	
	wt/*mbk-2*	10	1.79	0.69	0.22	0.75	0.29	0.09	<0.05
	glp-1(-)/control	10	2.39	0.35	0.11	1.00	0.15	0.05	
	glp-1(-)/*mbk-2*	10	4.01	1.14	0.36	1.68	0.48	0.15	<0.001

The effect of *mbk-2* knockdown on the expression of a *Psod-3::gfp* reporter gene *(muIs84)* relative to control-RNAi (vector L4440) treated animals was examined in wild-type and germline-less, *glp-1(-)* [*glp-1(e2144ts)*] worms. Fluorescence images were quantified, corrected for background, and fold-changes in reporter gene expression were calculated relative to wild-type and *glp-1(-)* animals. Statistical significance was determined by two-way ANOVA with Bonferroni post tests. Experiment #5 is shown in Supplementary Figure S2B.

```
DAF-16  242  TIETTTKAQLEKSRRGAKKRIKERALMGSLHSTL-NGNSIAGSIQTISHDLYDDDSMQGA  300
mFOXO1  254  -MDNNSKFAKSRGRAAKKKASLQSGQEGPGDSPGSQFSKWPASPGSHSND---------D  303
hFOXO1  257  -MDNNSKFAKSRSRAAKKKASLQSGQEGAGDSPGSQFSKWPASPGSHSND---------D  306
hFOXO3  254  -MDNSNKYTKSRGRAAKKKAALQTAPESADDSP-SQLSKWPGSPTSRSSD---------E  302
              ::...*   .:.* . **   : .  .  .*   : ..  .*  : * *

DAF-16  301  FDNVPSSFRPRTQSNLSIPGSSSRVSPAIGSDIYDDLE----------FPSWVGE-----  345
mFOXO1  304  FD-NWSTFRPRTSSNASTI--SGRLSPIMTEQDD--LGDG--DVHSLVYPPSAAK-----  351
hFOXO1  307  FD-NWSTFRPRTSSNASTI--SGRLSPIMTEQDD--LGEG--DVHSMVYPPSAAK-----  354
hFOXO3  303  LD-AWTDFRSRTNSNASTV--SGRLSPIMASTELDEVQDDDAPLSPMLYSSSASLSPSVS  359
             :*   : ** **.** *    *.*:** : .   :             :   ..

DAF-16  346  -----SVPAI-----------------PSDIVDRTDQ------MRIDATTHIGGVQIKQE  377
mFOXO1  352  --MASTLPSLSEISNPE---------NMENLLDNLNLLLSSPTSLTVSTQSSPGSMMQQTP  400
hFOXO1  355  --MASTLPSLSEISNPE---------NMENLLDNLNLLLSSPTSLTVSTQSSPGTMMQQTP  403
hFOXO3  360  KPCTVELPRLTDMAGTMNLNDGLTENLMDDLLDNIT-------LPPSQPSPTGGLMQRSS  412
                   :* :                  .:::*.         :  .  :  * :  :

DAF-16  378  SKPIK---TEPIAPPPSYHELNSVRGSCAQNPLLRNPIVPSTNFKPMPLPGAYGNYQNGG  434
mFOXO1  401  CYSFAPPNTSLNSPSPNYSK--YTYGQSSMSPLPQMPMQTLQDSKSS-----YGGLNQYN  453
hFOXO1  404  CYSFAPPNTSLNSPSPNYQK--YTYGQSSMSPLPQMPIQTLQDNKSS-----YGGMSQYN  456
hFOXO3  413  SFPYTTKGSGLGSPTSSFNS--TVFGPSSLNSLRQSPMQTIQENKPA----TFSSMSHYG  466
             .       :   :*  .: .   . * .: . * : *:    : *       :.. .: .
```

Supplementary Figure 1. NLK-sites in FOXO-proteins. ClustalΩ alignment of full-length DAF-16 with murine and human FOXO1 and human FOXO3. Only the part covering the 8 NLK-sites reported in murine FOXO1 is shown [6]. The Ser/Thr-residues phosphorylated by NLK are highlighted in blue, the obligatory Pro immediately following an NLK-phosphorylated Ser/Thr is highlighted in yellow. The only SP-site in this region that is conserved between DAF-16 and murine/human FOXO1s is Ser326/Ser326/Ser329. Note: NLK-phosphorylation of individual residues has been reported to be weak [6].

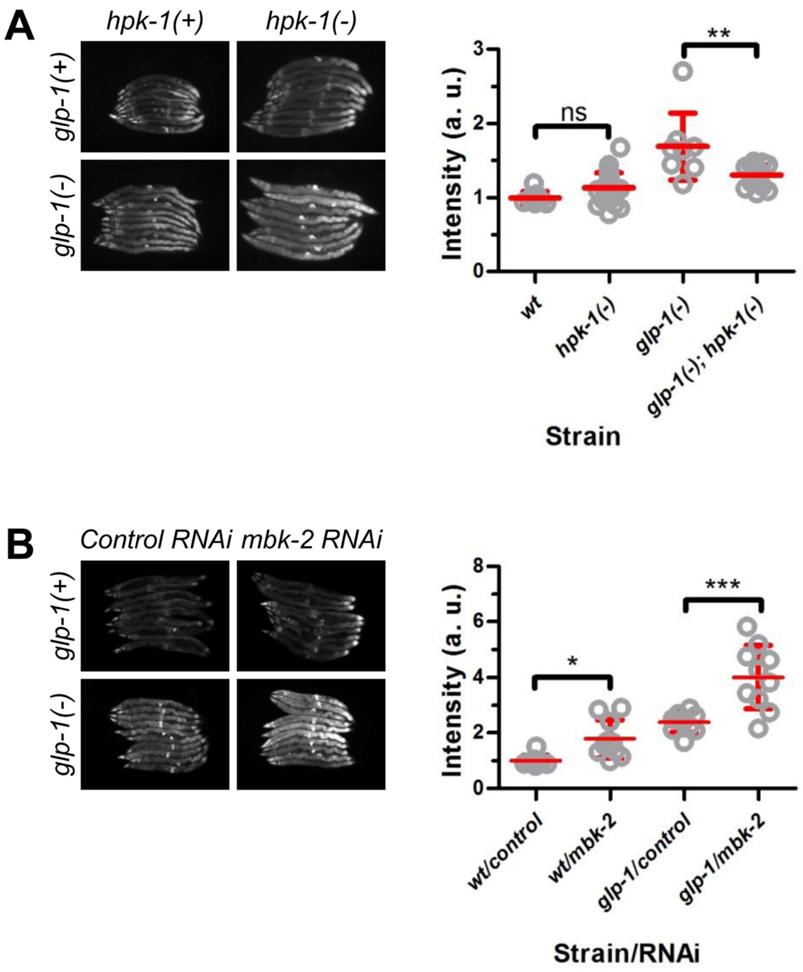

Supplementary Figure 2. Effect of DYRK-family kinases HPK-1 and MBK-2 on *Psod-3::gfp* expression. Accompanies Figure 3. (**A**) The *hpk-1* loss of function mutation *hpk-1(pk1393)* decreases *Psod-3::gfp*-expression in germline-deficient *glp-1(-)* [*glp-1(e2144ts)]*, but not in wild-type animals (representative experiment shown, n=5). (**B**) Depletion of *mbk-2* by RNAi increases *Psod-3::gfp*-expression in *glp-1(-)*, and –to a lesser extent- in wild-type background. RNAi treatment was initiated at the L1 stage (representative experiment shown, n=3. Error bars indicate standard deviations. Statistical significance of fluorescence intensity differences was determined by two-way ANOVA with Bonferroni post tests. All experiments in (**A**) and (B) were performed on day-2 adult worms. Images were taken at 100x magnification.

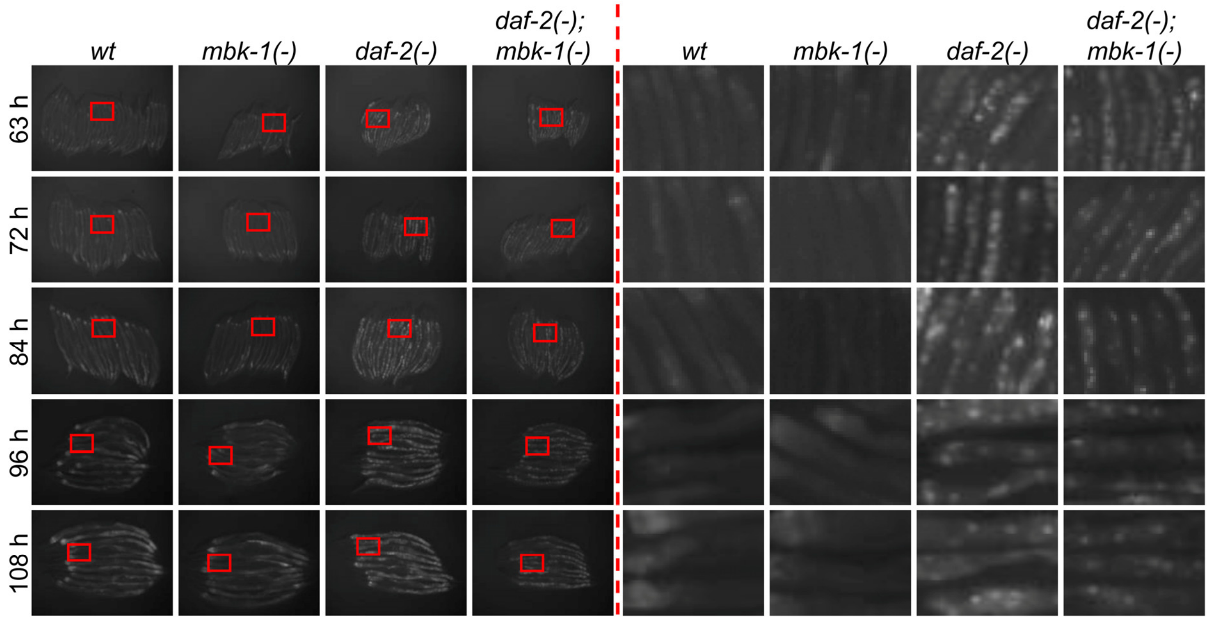

Supplementary Figure 3. Loss of *mbk-1* does not affect DAF-16 subcellular localization in *daf-2* mutant *C. elegans*. Accompanies Figure 4. The effect of the *mbk-1* loss of function mutation *mbk-1(pk1389)* on subcellular localization of an intestine-specific GFP::DAF-16 protein (encoded by transgene *muIs145[Pges-1::gfp::daf-16]*) was determined at the times indicated in wild-type and *daf-2(-)* [*daf-2(e1370)*] animals. Images on the left were taken at 100x magnification, images on the right are 6.5x magnifications of the areas boxed in red.
